# Zotatifin, an eIF4A-Selective Inhibitor, Blocks Tumor Growth in Receptor Tyrosine Kinase Driven Tumors

**DOI:** 10.3389/fonc.2021.766298

**Published:** 2021-11-24

**Authors:** Adina Gerson-Gurwitz, Nathan P. Young, Vikas K. Goel, Boreth Eam, Craig R. Stumpf, Joan Chen, Sarah Fish, Maria Barrera, Eric Sung, Jocelyn Staunton, Gary G. Chiang, Kevin R. Webster, Peggy A. Thompson

**Affiliations:** Department of Cancer Biology, eFFECTOR Therapeutics, Inc., San Diego, CA, United States

**Keywords:** zotatifin, eIF4A, PI3K, AKT, mTOR, receptor tyrosine kinase, RTK

## Abstract

Oncoprotein expression is controlled at the level of mRNA translation and is regulated by the eukaryotic translation initiation factor 4F (eIF4F) complex. eIF4A, a component of eIF4F, catalyzes the unwinding of secondary structure in the 5’-untranslated region (5’-UTR) of mRNA to facilitate ribosome scanning and translation initiation. Zotatifin (eFT226) is a selective eIF4A inhibitor that increases the affinity between eIF4A and specific polypurine sequence motifs and has been reported to inhibit translation of driver oncogenes in models of lymphoma. Here we report the identification of zotatifin binding motifs in the 5’-UTRs of HER2 and FGFR1/2 Receptor Tyrosine Kinases (RTKs). Dysregulation of HER2 or FGFR1/2 in human cancers leads to activation of the PI3K/AKT and RAS/ERK signaling pathways, thus enhancing eIF4A activity and promoting the translation of select oncogenes that are required for tumor cell growth and survival. In solid tumor models driven by alterations in HER2 or FGFR1/2, downregulation of oncoprotein expression by zotatifin induces sustained pathway-dependent anti-tumor activity resulting in potent inhibition of cell proliferation, induction of apoptosis, and significant *in vivo* tumor growth inhibition or regression. Sensitivity of RTK-driven tumor models to zotatifin correlated with high basal levels of mTOR activity and elevated translational capacity highlighting the unique circuitry generated by the RTK-driven signaling pathway. This dependency identifies the potential for rational combination strategies aimed at vertical inhibition of the PI3K/AKT/eIF4F pathway. Combination of zotatifin with PI3K or AKT inhibitors was beneficial across RTK-driven cancer models by blocking RTK-driven resistance mechanisms demonstrating the clinical potential of these combination strategies.

## Introduction

Receptor Tyrosine Kinases (RTKs) are integral membrane proteins with an extra-cellular ligand binding domain and a cytoplasmic kinase domain. RTKs are typically activated by ligand-induced dimerization, subsequently stimulating signal-transduction cascades in pathways that regulate key cellular processes, such as proliferation, survival and cell cycle control. The PI3K and RAS signaling pathways control these processes in response to extracellular cues (1, 2). Altered expression and/or dysregulation of RTKs that lead to their constitutive activation are associated with oncogenesis due to aberrant downstream signal transduction (3).

RTK stimulation of the PI3K and RAS signaling pathways converge on the eIF4F translation initiation complex, comprised of eIF4E cap-binding protein, eIF4G scaffolding protein and eIF4A DEAD box RNA helicase (4). The eIF4A-dependent translatome is enriched for mRNAs containing 5’-untranslated region (UTR) polypurine and GC rich sequence motifs with the potential to form structural elements (5–10). Zotatifin (eFT226), a selective eIF4A inhibitor, increases the affinity between eIF4A and specific polypurine sequence motifs in the 5’-UTR of zotatifin target genes, a mechanism of action similar to that of Rocaglamide A (8). The sequence specificity of zotatifin for a polypurine motif was demonstrated by direct binding studies using surface plasmon resonance (7). The formation of a ternary complex between zotatifin, eIF4A and mRNA blocks scanning of the pre-initiation 43S complex along the 5’-UTR leading to inhibition of protein expression for certain transcripts containing polypurine sequence motifs (6, 7). These polypurine motifs are enriched in the 5’-UTRs of known oncogenic drivers, including RTKs such as HER2 and FGFR1/2.

Here we show that zotatifin downregulates protein expression of FGFR1/2 and HER2 oncogenes, across a range of solid tumor models, resulting in potent anti-tumor activity and *in vivo* tumor growth inhibition and regression. We further identified that sensitivity to zotatifin in FGFR1/2 or HER2 driven tumor models is dependent on the activation state of the mTOR signaling pathway. While many drugs have been developed to target components of PI3K/AKT or RAS/ERK pathways as anticancer agents (11, 12), one well-characterized limitation of these targeted therapies is the rise of resistance mechanisms that involve RTK hyperactivation (1). We demonstrate that combination of zotatifin with PI3K or AKT inhibitors, which aim at vertical inhibition of the PI3K/AKT/eIF4F pathway, induce synergistic anti-tumor activity across many of these FGFR1/2 and HER2 driven tumors. We propose that the synergistic effect is obtained through sustained downregulation of RTKs together with PI3K/AKT pathway inhibition, thereby reducing the resistance mechanisms that limit the function of PI3K/AKT inhibitors.

## Materials and Methods

### Reagents

Zotatifin (eFT226) was prepared in-house (13). Alpelisib, Ipatasertib were purchased from MedChemExpress (NJ, USA). Cell lines were purchased from ATCC (VA, USA) (BT-474, Calu-3, HCC1419, HCC202, MDA-MB-361, MDA-MB-231, NCI-H1581, NCI-H716, NCI-N87, SNU-16, SK-BR-3, ZR-75-30), DSMZ (Braunschweig, Germany) (EFM-192A, JIMT-1, MFM-223) or MilliporeSigma (CA, USA) (OE19).

### Cell Proliferation Assay

Tumor cells were cultured in DMEM or RPMI media, 10% FBS and 1x penicillin/streptomycin. Exponentially growing cells were seeded at a density of 3,000-6,000 cells per well in 96-well white flat bottom polystyrene TC-treated plates (#29444-041, VWR, USA) in 90 µL growth media and cultured overnight. Cells were treated with zotatifin and the indicated compounds as single agents or in combination (fixed ratio) in a 10-point twofold dilution series. The final DMSO concentration was 0.1%. Cells were incubated for 72 h at 37°C in a CO_2_ incubator. Baseline viability of untreated cells was measured on the day of treatment and proliferation was measured after 72 h of drug treatment using CellTiter-Glo (CTG) reagent (Promega, WI, USA) according to manufacturer’s instructions. Calculation of CTG % Inhibition = [([Inhibitor] – baseline)/(DMSO – baseline)] x 100. The calculated signals were plotted using GraphPad Prism software.

### Apoptosis Induction Assays

For Caspase 3/7 activation detection (Promega, WI, USA), cells were seeded and treated with identical conditions to those described for the cell proliferation assay. For analysis, data was first normalized against a viability measurement using CellTiter-Glo (see above) and then normalized to the signal in control (DMSO) wells.

For Annexin V detection, cells were seeded in 96-well round bottom non-tissue culture treated plates at a density of 20,000 cells per well, and then cultured overnight. BD Annexin V FITC Apoptosis Detection Kit was used (BD Biosciences, CA, USA). Briefly, cells were washed with PBS and resuspended in 1X Annexin V binding buffer containing Annexin V and Propidium Iodide (PI). Cells were incubated at room temperature for 15 min followed by dilution in Annexin V buffer. Fluorescence of the cells was monitored by flow cytometry using Attune NxT flow cytometer (ThermoFisher Scientific, MA, USA). Data was plotted using GraphPad Prism software.

### 5’-UTR Luciferase Reporter Assays

Sequences representing the 5’ untranslated regions (UTRs) of TUBA, FGFR1, FGFR2 and HER2 (**Supplementary Table S1**) were synthesized and cloned upstream of firefly luciferase into the Nhe I and Hind III restriction endonuclease sites of pGL4-CMV-T7 (Genewiz, NJ, USA). DNA templates were linearized by digestion with Bam HI (NEB, MA, USA) and purified with the QIAquick PCR Purification kit (QIAGEN, MD, USA) according to manufacturer’s instructions. Templates were *in vitro* transcribed using the mMessage mMachine T7 ULTRA transcription kit (Ambion, ThermoFisher Scientific, MA, USA) and purified using the MEGAclear kit according to manufacturer’s instructions. HEK-293t cells were seeded at 40,000 cells/well in 96 well plates 24 h prior to transfections. 64 µL of fresh complete media (DMEM, 10% FBS, antibiotics) was added prior to transfections. RNA transfections were carried out using the TransIT-mRNA transfection kit (Mirus Bio, WI, USA) according to manufacturer’s instructions and 8 µL transfection mix was added to each well. Transfected cells were incubated for 30 min prior to adding an additional 8 µL of media containing varying concentrations of zotatifin. Cells were further incubated for 4 h in the presence of drug prior to analyzing luciferase levels using the ONE-Glo luciferase assay system (Promega, WI, USA) according to manufacturer’s instructions. Relative luciferase signal was background subtracted and normalized to DMSO treated controls prior to data analysis and plotting in Prism software (GraphPad, CA, USA).

### Polysome Profiling

Cells were seeded in 10 cm dishes at 5x10^6^ cells per dish and cultured overnight and were then treated with 20 nM zotatifin or DMSO for 3 h. Cells were treated with 0.1 mg/mL cycloheximide for 5 min before lysis, washed in ice-cold PBS plus 0.1 mg/mL cycloheximide and lysed in polysome lysis buffer (20 mM Tris-HCl pH 7.4, 150 mM NaCl, 5 mM MgCl_2_, 10% Triton X-100, 1 mM DTT, DNase I (2U/µL), SUPERase•In RNase Inhibitor (20U/µL), 0.1 mg/mL cycloheximide, 10% NP-40). Lysates were clarified by centrifugation for 10 min at 20,000 x g at 4°C. RNA concentration was quantitated using Ribogreen. 10-50% gradients were prepared using the Gradient Master (BioComp Instruments, Fredericton, Canada). Gradients were centrifuged in an SW-41Ti rotor at 40,000 rpm at 4°C for 2 h and then fractionated using a Gradient Station (BioComp Instruments, Fredericton, Canada). 5 ng of luciferase mRNA was added to each fraction for normalization. RNA was extracted from each fraction using TriZol and transcript abundance determined by quantitative PCR (qPCR) using SYBR Green PCR mix and primers specific for each transcript. Measurements were normalized to luciferase abundance and plotted as percent detected.

### Preparation of Lysates and Western Blot Analysis

Exponentially growing cells were seeded at 1-2x10^6^ cells per well in 6-well plates. Cells were treated the next day with DMSO, zotatifin (eFT226), Alpelisib, Ipatasertib or combinations for 24 h. Cells were lysed in 1X RIPA lysis buffer (EMD Millipore, MA, USA) supplemented with 1X Protease and Phosphatase inhibitors (Bimake.com, Tx, USA), gently scraped off the plates and collected into microfuge tubes. Tubes were centrifuged for 15 min at 15,000 RPM, and supernatant was collected. Protein concentrations in lysates were quantitated by BCA protein assay (ThermoFisher Scientific, MA, USA). Equal amounts of total protein were resolved by SDS-PAGE, immunoblotted with the indicated antibodies, and visualized by LI-COR Odyssey imager (LI-COR, NE, USA).

Primary antibodies: AKT CST#2920; AKT CST#9272; p-AKT S473 CST#4060; HER2 CST#2248; rpS6 CST#2317; rpS6 CST#2217; p-rpS6 S240/244 CST#5364; p-rpS6 S235/236 CST#4858; p70S6k CST#2708; p-p70S6k T389 CST#9234; ERK1/2 CST#4696; p-ERK1/2 T202/Y204 CST#4370; eIF4B CST#13088; p-eIF4B S406 CST#8151; p-eIF4B S422 CST#3591; 4EBP CST#9644; p-4EBP S65 CST#9456; PDCD4 CST#9535; Beta actin CST#3700; Beta tubulin CST#86298; PRAS40 CST#2691; p-PRAS40 T246 CST#13175; ER alpha CST#8644; FGFR2 CST#11835; HER3 #12708; p-HER3 #2842; FoxO3a #2497; p-FoxO1 (T24)/FoxO3a (T32) #9464 (Cell Signaling Technology, MA, USA), Cyclin D1 #241R-45 (MilliporeSigma, CA, USA), GAPDH (Santa Cruz Biotechnology, TX, USA).

Secondary antibodies: Donkey anti-rabbit IRDye-800CW, Donkey anti-mouse IRDye-680RD for Odyssey infrared imaging (LI-COR, NE, USA).

### Nascent Protein Synthesis Assay

Nascent protein synthesis assay was preformed using Click-iT^®^ Plus OPP Alexa Fluor^®^ 594 Protein Synthesis Assay Kit (#C10457, ThermoFisher Scientific, USA) according to the manufacturer’s instructions. Exponentially growing cells were seeded at 50,000-100,000 cells per well were seeded in 24-well plates. Cells were treated the next day with DMSO or zotatifin for indicated periods of time. O-propargyl-puromycin (OPP) was added 30 min prior to sample collection. For determination of background signal of Alexa Fluor^®^ 594, samples where no OPP was added were processed.

Prior to fixation, cells were washed with phosphate-buffered saline (PBS), harvested from wells with Trypsin-EDTA and washed again with PBS. Cells were then fixed with formaldehyde-based fixation buffer (#420801, Biolegend, USA) for 15 min at room temperature. Cells were then washed twice with PBS, 4% FBS, 1 mM EDTA and incubated overnight at 4°C. Samples were transferred to a 96-well plate, cells were permeabilized with 0.5% Triton X-100 in PBS for 15 min at room temperature, then washed with PBS, 4% FBS, 1 mM EDTA and stained for 30 min with Click-iT Plus OPP reaction cocktail prepared based on kit’s protocol. Cells were washed with PBS, 4% FBS, 1 mM EDTA and screened by flow cytometry using Attune NxT flow cytometer (ThermoFisher Scientific, MA, USA). Data were analyzed by FlowJo software and plotted using Prism software (GraphPad, CA, USA).

### *In Vivo* Studies

All animal studies were carried out in accordance with the guidelines established by the Institutional Animal Care and Use Committee at Explora BioLabs (San Diego, CA, ACUP# EB17-010-033), Crown Biosciences (Beijing, China) or Wuxi AppTec (Shanghai, China). For subcutaneous xenograft studies, mice (6-8-week-old females, 16-24 grams) were implanted with an equal volume (1:1) ratio of tumor cells and Matrigel (BD Biosciences, CA, USA) for tumor development. When the mean tumor size reached ~100-200 mm^3^, the mice were randomized and size-matched into vehicle and treatment groups. Tumor size was measured in length and width with a caliper twice a week. The tumor volume was calculated by the formula LxWxW/2 according to NCI standards. Body weights were collected prior to study start and twice a week during the study. Zotatifin was formulated in 5% dextrose in water (D5W) and immediately dissolved into solution and administered IV Q4D.

BT-474 - 1x10^7^ cells in athymic nude ((NCr) nu/nu fisol, Simonsen), mean tumor initiation size 183 mm^3^, 9 animals/group; MDA-MB-361 - 1x10^7^ cells in NOD.SCID (Charles River, Hollister, CA), mean tumor initiation size 158 mm^3^, 9 animals/group; ZR-75-30 - 1x10^6^ cells in athymic nude, mean tumor initiation size 266 mm^3^, 10 animals/group; JIMT-1 - 5x10^6^ cells in SCID Beige, mean tumor initiation size 163 mm^3^, 9 animals/group; SNU-16 - 1x10^7^ cells in BALB/c nude, mean tumor initiation size 147 mm^3^, 8 animals/group; OE19 - 5x10^6^ cells in BALB/c nude, mean tumor initiation size 153 mm^3^, 8 animals/group; Calu3 - 1x10^7^ cells in BALB/c nude, mean tumor initiation size 140 mm^3^, 8 animals/group.

For the orthotopic xenograft model MDA-MB-231, athymic nude ((NCr) nu/nu fisol, Simonsen Laboratories, CA, USA) were implanted orthotopically in the mammary fat pad with 5x10^6^ cells in 0.1 mL of un-supplemented DMEM and an equal volume (1:1) ratio of Matrigel (BD Biosciences, CA, USA); mean tumor initiation size was 201 mm^3^, 10 animals/group.

In drug combination studies, 5x10^6^ of JIMT-1 cells were implanted in SCID Beige mice, mean tumor initiation size 161 mm^3^, 9 animals/group; MFM-223(PDX) tumor fragments (passage 2, P2) were implanted in athymic nude mice, mean tumor initiation size 382 mm^3^, 7 animals/group.

Statistical analysis of difference in tumor volume among the groups were conducted on the data obtained at the last day of treatment and subsequently evaluated using the one-way ANOVA, no matching and corrected for multiple comparisons using Dunnett's t-test (equal variance assumed). All data were analyzed using GraphPad Prism, where p < 0.05 was considered as statistically significant.

## Results

### Zotatifin (eFT226) Downregulates Receptor Tyrosine Kinase Protein Levels

**Figure 1:**
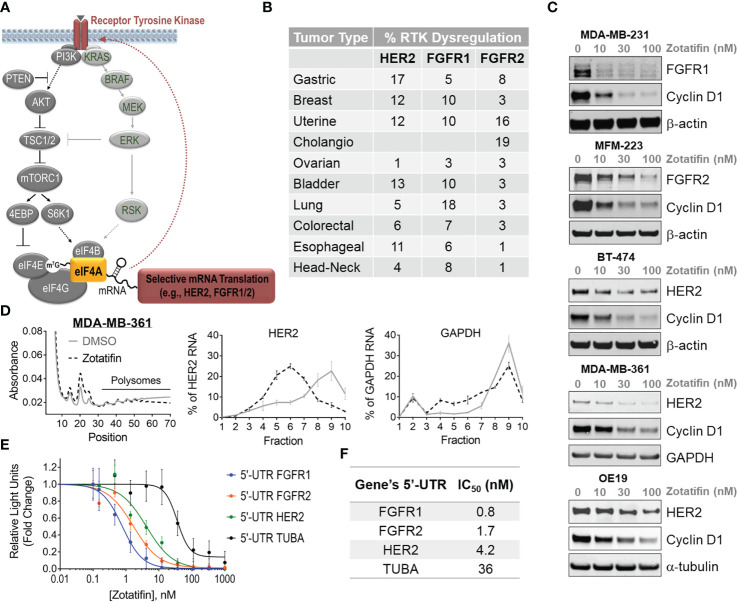
Zotatifin translationally downregulates FGFR1/2 and HER2 in RTK-driven tumors. **(A)** Schematic of RTK-stimulated PI3K/AKT and RAS/ERK pathways that activate eIF4A. **(B)** Frequency of HER2 and FGFR1/2 dysregulation in different cancer types. **(C)** Western blot analysis of RTKs and cyclin D1 levels following 24 h treatment with zotatifin or DMSO in HER2- or FGFR1/2-driven tumor models. β-actin or α-tubulin serve as loading controls. **(D)** Polysome profiling and mRNA distribution in MDA-MB-361 breast cancer cells treated for 3 h with 20 nM zotatifin compared to DMSO. mRNA levels of HER2 are monitored in polysome fractions. GAPDH serves as a control. **(E)** Luciferase reporter assay with gene constructs containing 5’-UTRs of FGFR1/2, HER2 or TUBA in HEK293 cells following 4 h of dose-dependent treatment with zotatifin. **(F)** Left, list of genes of which their 5’-UTR sequences were cloned into luciferase reporter constructs. Right, measured IC_50_ values (nM) for inhibition of expression following 4 h treatment with zotatifin.

eIF4A integrates signals from two major RTK signaling pathways, PI3K and RAS, which regulate cell growth, proliferation, survival and apoptosis (**Figure 1A**). This creates a context whereby RTK alterations activate eIF4A with the potential to become hypersensitive to eIF4A inhibition. The RTKs HER2 and FGFR1/2 are dysregulated through mutations, amplifications or fusions and are driver oncogenes in a wide range of cancers [**Figure 1B**, (3)]. Treatment of RTK-driven cancer cell lines with zotatifin for 24 h resulted in dose dependent downregulation of HER2, FGFR1 and FGFR2 protein levels, as well as cyclin D1 (a zotatifin target gene (6), **Figure 1C**, **Supplementary Figure S1A**).

Translational downregulation of FGFR1/2 and HER2 oncogenes by eIF4A inhibitors has previously been identified by genome wide ribosome profiling following treatment with either silvestrol or rocaglamide A (8, 9). To better understand the mechanism of RTK downregulation by zotatifin, we evaluated the mRNA distribution in polysome fractions in HER2^amp^ (**Figure 1D**) and FGFR2^amp^ (**Supplementary Figure S1B**) cell lines (MDA-MB-361 (breast) and NCI-H716 (colorectal), respectively) with or without 20 nM zotatifin treatment. We observed that zotatifin significantly inhibited the translation of HER2 and FGFR2 as seen by a decrease in mRNA polysome occupancy compared to control mRNAs (GAPDH or POLR2). This observation confirmed that these RTKs are translationally downregulated by zotatifin.

We further showed that the UTRs of HER2 and FGFR2 are essential for the downregulation by zotatifin. Treatment of MDA-MB-361 (HER2^amp^) and SNU-16 (FGFR2^amp^) cell lines with 25-30 or 100 nM zotatifin for 24 h resulted in dose dependent downregulation of the endogenous HER2 and FGFR2 protein levels as analyzed by western analysis (**Supplementary Figure S1C**). In contrast, overexpression of constructs lacking the RTK UTR (ΔUTR) were refractory to downregulation by zotatifin (HER2(ΔUTR) in MDA-MB-361 and FGFR2(ΔUTR) in SNU-16 cells). A decrease in cyclin D1 protein levels confirmed zotatifin activity in all conditions tested. The housekeeping genes (*i.e*. α-tubulin, β-actin, vinculin or GAPDH) were unchanged in both parental and ΔUTR cell lines (**Supplementary Figure S1C**). These observations are consistent with our earlier reports that zotatifin translationally downregulates its target genes through polypurine RNA motifs within the 5’-UTR, which act as potential selective interaction sites for zotatifin-induced ternary complex formation between eIF4A, RNA and zotatifin (6, 7).

Sequence analysis of the 5’-UTR of FGFR1, FGFR2 and HER2 identified polypurine elements similar to the high affinity motifs identified in the direct binding studies. To test whether zotatifin-dependent translational regulation of FGFR1, FGFR2 and HER2 mRNAs is mediated through their 5’-UTR, HEK293t cells were transiently transfected with luciferase reporter constructs containing the 5’-UTR of each RTK or TUBA. We found that treatment with zotatifin inhibited translation of each reporter construct in a dose dependent manner (**Figure 1E**). zotatifin was ~10-45 fold more potent at inhibiting expression of the luciferase reporter gene encoded by constructs containing an RTK 5’-UTR (IC_50_ of 0.8-4.2 nM) *versus* the TUBA 5’-UTR which has no polypurine motif (IC_50_ = 36 nM) (**Figure 1F**). Our findings support the notion that zotatifin’s mechanism of translational regulation is dependent on polypurine motifs within the 5’-UTR resulting in the downregulation of RTK protein levels. These data further highlight the unique circuitry generated by the RTK-driven signaling pathway, where RTK activation stimulates the activity of eIF4A through phosphorylation of its regulators (14, 15), while eIF4A inhibition by zotatifin downregulates RTK translation (**Figure 1A**).

### Zotatifin Promotes Anti-Tumor Activity Across RTK-Driven Solid Tumor Cancers

**Figure 2:**
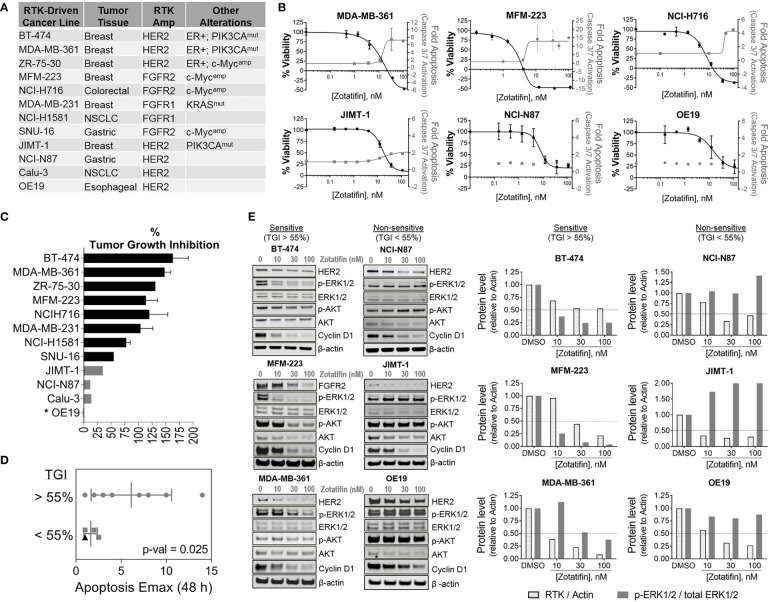
Anti-tumor activity of zotatifin in RTK-driven cancer models **(A)** List of cell lines used with their associated amplified RTK drivers, tissue of origin and additional oncogenic alterations. **(B)** Dose dependent proliferation (black) and apoptosis (grey) response curves with *in vitro* zotatifin treatment for 48 h **(C)** Percent tumor growth inhibition (% TGI) of xenograft studies following treatment with 1 mg/kg zotatifin (0.1 mg/kg for OE19, marked with asterisk). Black, TGI > 55%; Grey, TGI < 55%. **(D)** *In vitro* Apoptosis Emax values obtained after 48 h treatment with zotatifin grouped based on their *in vivo* sensitivity to zotatifin. TGI, tumor growth inhibition (see **Figure 2C**) with 1 mg/mL zotatifin (0.1 mg/kg for OE19, marked with black triangle). P-value is shown. **(E)** Left, western blot analysis of RTK, Cyclin D1, AKT, ERK1/2, p-AKT S473 and p-ERK1/2 T202/Y204, in zotatifin-sensitive *versus* non-sensitive cell lines. β-actin serves as loading control. Right, analysis of indicated protein levels.

Given that zotatifin downregulates the protein expression of RTK oncogenic drivers (**Figure 1C** and **Supplementary Figure S1**), the anti-tumor activity of zotatifin (*i.e*., effect on proliferation and apoptosis) was tested in a panel of cancer cell lines that are driven by alterations in FGFR1/2 and HER2 (**Figure 2A**). Treatment with zotatifin *in vitro* resulted in significant dose dependent inhibition of proliferation as well as induction in apoptosis as monitored by activation of Caspase-3/7 in most of the cell lines tested (**Figure 2B** and **Supplementary Figure S2A**). These results are consistent with zotatifin’s downregulation of RTK oncogenic drivers as well as the cell cycle regulatory protein cyclin D1.

To validate the *in vitro* anti-tumor activity of zotatifin and explore whether proliferation inhibition or induction of apoptosis may predict response to zotatifin *in vivo*, xenograft models derived from the RTK-driven tumor cell lines were developed. The tumor models were grown in immune compromised mice and treated with zotatifin or vehicle administered I.V. on a Q4D schedule. Zotatifin treatment resulted in significant tumor growth inhibition (TGI > ~60%) (16) and regression across a broad set of RTK-driven tumor models of breast, colorectal, lung and gastric (**Figure 2C** and **Supplementary Figure S2B**). Administration of 1 mg/kg zotatifin resulted in significant tumor growth inhibition in eight out of the twelve models tested (“zotatifin sensitive”: BT-474, MDA-MB-361, ZR-75-30, MFM-223, NCI-H716, MDA-MB-231, NCI-H1581 and SNU-16). With the same dose of zotatifin (1 mg/kg), no significant tumor growth inhibition (TGI < 55%) was observed in three other models, termed “zotatifin non-sensitive” (JIMT-1, NCI-N87 and Calu-3) (**Figure 2C**). Based on tumor growth inhibition data with 0.1 mg/kg zotatifin (**Supplementary Figure S2C**), we consider OE19 as a non-sensitive model as well. Comparing the anti-tumor activity, we found that fold induction of apoptosis after 48 h treatment with zotatifin *in vitro* (“Apoptosis Emax”) was a strong predictor of sensitivity to zotatifin *in vivo*, as six out of eight sensitive lines exhibited an “Apoptosis Emax” ≥ 3, while all three non-sensitive lines exhibited lower levels of apoptosis with “Apoptosis Emax” ≤ 2 (**Figure 2D**).

Zotatifin’s effect on RTK protein levels was further evaluated in the FGFR1 and FGFR2 driven xenograft models, NCI-H1581 (FGFR1^amp^) and NCI-H716 (FGFR2^amp^). Tumor samples were collected 24 h post the last dose of zotatifin (NCI-H716, 14 days treatment; NCI-H1581, 20 days treatment). Durable inhibition of RTK expression in tumor tissue following treatment with zotatifin (**Supplementary Figure S2D**) is consistent with the significant tumor growth inhibition observed in these models (**Figure 2C**), indicating that monitoring RTK protein levels can be a pharmacodynamic marker of response.

In all models tested *in vitro*, zotatifin treatment resulted in the common effect of FGFR1/2 or HER2 downregulation (**Figure 1C** and **Supplementary Figure S1A**), yet some models were more sensitive to zotatifin than others (**Figure 2C**). To further characterize the differential effects of zotatifin on survival of tumors, the impact of zotatifin on key signaling proteins within the PI3K and/or RAS pathways were evaluated across a panel of zotatifin-sensitive and non-sensitive RTK-driven tumor cell lines. Protein levels of AKT (total and p-AKT S473), and ERK (total and p-ERK1/2 T202/Y204) were evaluated following 24 h treatment with zotatifin. It became evident that while treatment with zotatifin decreased tested RTKs levels in all models, the levels of downstream effectors (*i.e*. p-ERK as well as p-AKT) were decreased only in the sensitive models (**Figure 2E**), suggesting that regulation of MAPK or AKT signaling pathways downstream of RTKs may be a predictor of response.

### Sensitivity to Zotatifin in RTK-Driven Tumors Is Correlated With Basal Levels of mTOR Pathway Activity

**Figure 3:**
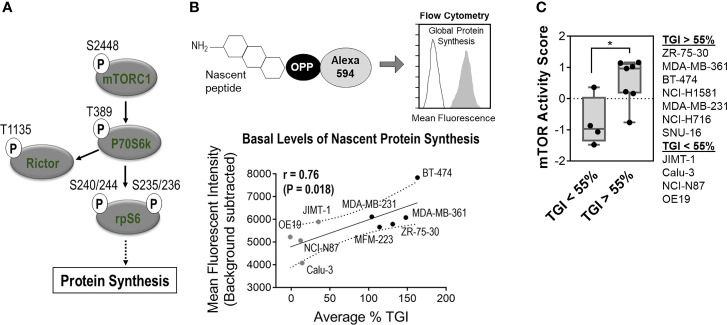
Sensitivity to zotatifin is correlated with basal level of mTOR activity. **(A)** Simplified schematic of components in mTOR signaling pathway used for “mTOR activity” score calculation (**Supplementary Figure S3**). Schematic highlights mTOR activity role in driving protein synthesis. **(B)** Flow-cytometry based measurement of basal levels of nascent protein synthesis as a function of percent of tumor growth inhibition with 1 mg/kg zotatifin (0.1 mg/kg for OE19). Levels of nascent protein synthesis are represented by mean fluorescence intensity in cells labeled with O-propargyl-puromycin (OPP) and Alexa 594. P, P_value_ (t-test); r, Pearson correlation. (See % TGI in **Figure 2C**). **(C)** Analysis of calculated “mTOR Activity” score of depicted RTK-driven tumors, as a function of tumor growth inhibition (% TGI) measured for these tumors.

mTOR signaling stimulates translation initiation and protein synthesis [(17), **Figure 3A**]; therefore, we were interested in evaluating whether differences in mTOR pathway activation correlated with sensitivity to zotatifin across cell lines. Nascent protein synthesis was monitored by O-propargyl-puromycin (OPP) labeling of cells across a panel of cell lines. We found that basal levels of nascent protein synthesis positively correlated with the *in vivo* activity observed for these tumor models treated with zotatifin (**Figure 3B**).

A bioinformatic analysis of protein as well as signaling markers was conducted using the CCLE RPPA database (18). Strikingly, markers that positively correlated with sensitivity were also related to activation of mTOR signaling. These include p-mTOR (S2448), p-p70S6K (T389), p-Rictor (T1135), and p-rpS6 (S240/244 and S235/236) (**Supplementary Figure S3**). A composite “mTOR activity” score, determined from the average distribution score of these phosphorylation biomarkers, was grouped based on *in vivo* tumor growth inhibition (**Figure 3C**). This analysis shows that tumor models with high mTOR activity corresponded to greater tumor growth inhibition with zotatifin treatment, a dependence that was also observed for zotatifin in a panel of lymphoma models (6).

Tumors with constitutive activation of the translation initiation machinery are hypothesized to become addicted to, and dependent on, protein synthesis for their maintenance (19). Since inhibition of eIF4A by zotatifin downregulates oncogenic drivers that facilitate this addiction, we anticipated that tumors with higher translation activity would be more sensitive to zotatifin. Indeed, of the tested tumor models, the zotatifin-sensitive models were those with higher “mTOR activity” scores and higher basal levels of nascent protein synthesis (**Figures 3B, C**). Together, these findings highlight the dependency of zotatifin-sensitive cell lines on mTOR signaling and translation.

### Vertical Inhibition of the PI3K-AKT-eIF4F Pathway Is Synergistic in RTK-Driven Solid Tumors

It is well documented that PI3K/AKT inhibitor-based treatments are often limited by intrinsic and acquired resistance mechanisms associated with hyper-activation of upstream nodes in the PI3K/AKT pathway, that can be attributed in part to RTK hyperactivation (1, 20, 21). Considering that zotatifin translationally downregulates the protein level of select RTKs (**Figures 1C–F** and **Supplementary Figure S1**), a rational combination strategy included combining zotatifin with PI3K/AKT pathway inhibitors. We hypothesized that vertical pathway inhibition could provide a means to overcome resistance mechanisms associated with RTK activation seen with PI3K/AKT targeted therapies.

**Figure 4:**
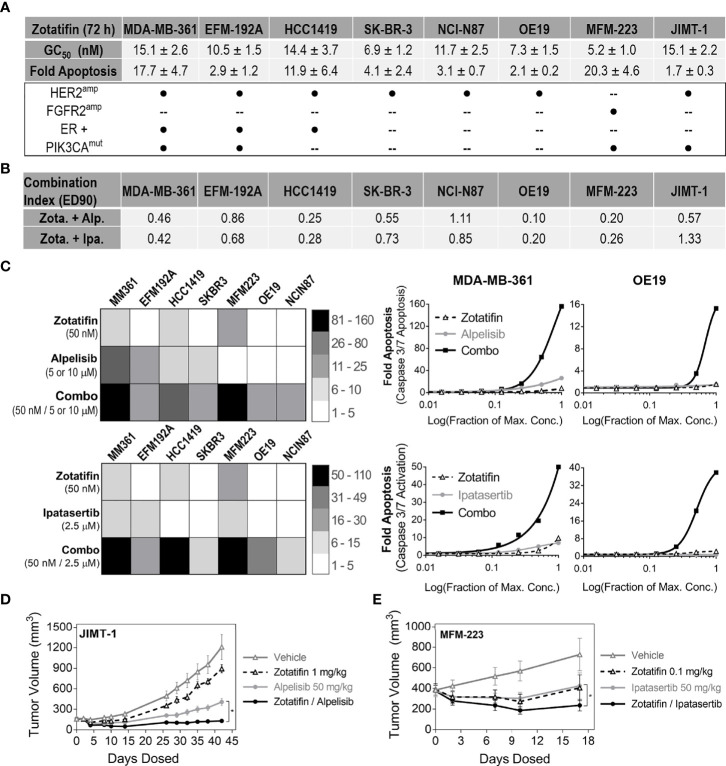
Combination of zotatifin with PI3K/AKT inhibitors demonstrate synergistic anti-tumor activity across RTK-driven tumors. **(A)** List of RTK-driven tumors used for combination treatments and their associated driver mutations. Presented for each tumor model are GC_50_ values following 72 h treatment with zotatifin and maximal fold apoptosis induced by 100 nM zotatifin. **(B)** Combination index values at 90% effective dose (ED_90_) for *in vitro* combination treatments of zotatifin with either Alpelisib (PIK3CAi) or Ipatasertib (AKTi). **(C)** Left, heat map representations of fold induction of apoptosis (Caspase 3/7 activation relative to DMSO) at indicated concentrations of single agents or combinations. Right, examples of synergistic induction of apoptosis (Caspase 3/7 activation relative to DMSO) in two different lines, MDA-MB-361 and OE19. For JIMT-1, both combination treatments yielded a 2-fold greater induction of apoptosis compared to the single agent alone (1.1-1.3 fold). Abbreviated names of the RTK-driven cell lines analyzed are depicted on top of each heat map. (Alpelisib, maximal concentration 10 μM with OE19 or HCC1419, 5 μM with other models; Ipatasertib, maximal concentration 2.5 μM; zotatifin, maximal concentration 50 nM). **(D)** SCID Beige mice bearing JIMT-1 xenografts treated with vehicle, zotatifin (1 mg/kg, Q4D), Alpelisib (50 mg/kg, QD) or combination therapy over 40 days. **(E)** SCID Beige mice bearing MFM-223 xenografts treated with vehicle, zotatifin (0.1 mg/kg, Q4D), Ipatasertib (50 mg/kg, QD) or combination over 17 days. Shown are average tumor volumes as measured during the course of treatment *p-value < 0.05.

A panel of RTK-driven lines (**Figure 4A**) were selected for combination treatment of zotatifin with inhibitors aimed at blocking PI3K/AKT signaling pathway; Alpelisib (PIK3CA inhibitor), approved by the FDA for treatment of metastatic breast cancer (22), or Ipatasertib (AKT inhibitor) (23), currently under phase III clinical evaluation. The panel of cell lines was designed to include breast cancer models that were ER positive and/or had PIK3CA mutations (in addition to the amplified driver RTK) as these alterations are common in RTK-driven cancers. Cells were treated for 72 h with each agent alone or in combination with zotatifin, using a 2-fold drug dilution series. Maximal drug concentrations of all agents were designed to be ~10-fold higher than their GC_50_ value for proliferation in each cell line. Drug combination benefit was analyzed using CalcuSyn to determine combination index values (CI) based on proliferation measurements, where synergy is defined as CI < 0.9, additive activity 0.9-1.1 and antagonism > 1.1 (24). Combination with these targeted agents demonstrated additive to synergistic inhibition of proliferation across the panel of cell lines (**Figure 4B**).

Since the extent of apoptosis was a strong predictor for *in vivo* tumor growth inhibition by zotatifin (**Figure 2D**), we further measured fold induction of apoptosis (by caspase 3/7 activation) relative to control DMSO for all combination treatments. Measurements of fold induction of apoptosis at maximal concentrations, summarized by heat maps (**Figure 4C**), highlight the benefit that combining zotatifin with PI3K or AKT inhibitors has on induction of tumor cell death in the RTK-driven cell lines examined.

The combination benefit was also evaluated *in vivo* using RTK-driven xenograft models. The HER2^amp^ JIMT-1 xenograft model was treated over 40 days with zotatifin (1 mg/kg, Q4D) or Alpelisib (50 mg/kg, QD) as single agents that resulted in 30% and 76% tumor growth inhibition, respectively. Combination treatment, however, resulted in significantly enhanced tumor growth inhibition and regression (103% TGI) (**Figure 4D**), consistent with the synergistic anti-proliferative activity determined *in vitro*. For the MFM-223 FGFR2^amp^ xenograft model, treatment for 17 days with zotatifin (0.1 mg/kg, Q4D) or Ipatasertib (50 mg/kg, QD) as single agents resulted in 96% and 86% tumor growth inhibition, respectively. Combination treatment for these two agents also resulted in significant tumor regression (140% TGI) (**Figure 4E**), in agreement with the increase in apoptosis activity observed *in vitro* (**Figure 4C**). All treatment conditions were well tolerated as seen by a lack of body weight loss (**Supplementary Figures S4A, B**). The significantly enhanced anti-tumor activity observed across a diverse set of RTK-driven tumor models treated with combinations that vertically inhibit the PI3K/AKT/eIF4F pathway suggest that combination of zotatifin with PI3K or AKT inhibitors can result in enhanced activity for these targeted agents.

### Combination With Zotatifin Suppresses Feedback Relief Induced by PI3K/AKT Inhibition

In HER2-amplified breast cancers, overexpression of HER2 dysregulates PI3K/AKT signaling by promoting HER2-HER3 heterodimer formation and HER3 signaling activation (25). Activation of the PI3K/AKT pathway by RTK overexpression or amplification is balanced by negative feedback mechanisms, such as the inhibitory phosphorylation of FOXO transcription factors by AKT (26). Intrinsic and acquired resistance to PI3K/AKT inhibitors has been attributed to relief of the negative feedback, which in turn leads to induction of FOXO-driven transcription and activation of RTKs, such as HER3, or to stimulation of other compensatory activation mechanisms of the PI3K/AKT pathway (27). The resulting enhanced compensatory PI3K/AKT activity limits the anti-tumor activity of PI3K/AKT inhibitors (21). Based on this, we speculated that the synergistic anti-tumor activity obtained by combination of zotatifin with PI3K/AKT inhibitors was due, at least in part, to a more penetrant elimination of RTK induction, thus reversing the outcome of feedback relief.

To evaluate the hypothesized mechanism that combination treatments may reverse the outcome of feedback relief by PI3K/AKT inhibitors, the signaling levels of HER3, AKT and AKT substrates, FOXO3a or PRAS40, were monitored in cells treated with either zotatifin, PI3K or AKT inhibitors alone or in combination. The protein level of cyclin D1 was also monitored as a marker for zotatifin activity as well as an additional measure of effects on cancer cell proliferation (28, 29).

**Figure 5:**
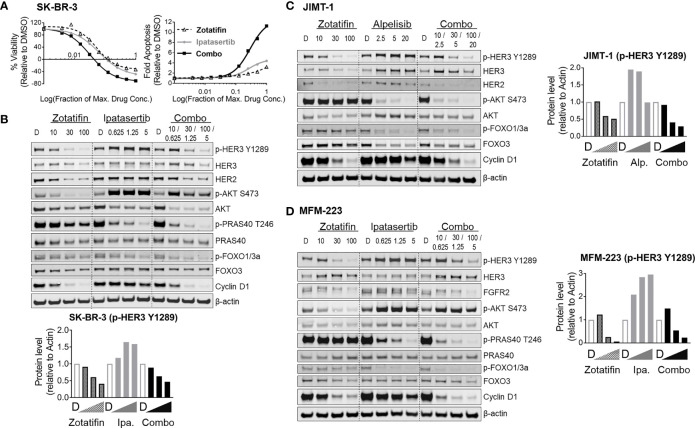
Zotatifin downregulates RTK levels induced by PI3K/AKT inhibition feedback *in vitro*. **(A, B)** SK-BR-3 HER2^amp^ cell line treated with zotatifin and Ipatasertib (AKTi); **(A)** Proliferation and apoptosis induction curves following 72 h of single agent or combination treatments (see also **Figures 4B, C**). **(B)** Western blots analysis following 48 h treatment with single agents or combinations. **(C)** JIMT-1 HER2^amp^ cell line treated with zotatifin and Alpelisib (PIK3CAi) for 24 h; **(D)** MFM-223 FGFR2^amp^ cell line treated with zotatifin and Ipatasertib (AKTi) for 24 h; β-actin or GAPDH serve as loading controls. Quantification of p-HER3 Y1289 protein levels is shown for each condition. Concentrations of drugs used are indicated on top of each blot: Ipa., Ipatasertib (µM); Alp., Alpelisib (µM); zotatifin (nM); D, DMSO.

The mechanism of beneficial anti-tumor activity for zotatifin combined with Ipatasertib (AKT inhibitor) was evaluated in the HER2 driven breast cancer SK-BR-3 model (**Figures 4B, C**, **5A**), that had previously shown upregulation of HER3/p-HER3 upon treatment with an AKT inhibitor (20). Ipatasertib, an ATP competitive inhibitor, has been shown to increase phosphorylation of AKT due to stabilization of a conformation that is inaccessible to phosphatases (30). Therefore, to examine AKT inhibition following treatment with Ipatasertib, the phosphorylation of AKT substrates p-PRAS40 and p-FOXO1/3a were monitored. As expected, increased AKT phosphorylation along with reduced PRAS40 and FOXO1/3a phosphorylation in SK-BR-3 cells were observed, confirming AKT inhibition by Ipatasertib (**Figure 5B**). Ipatasertib treatment of SK-BR-3 cells was also observed to induce HER3 phosphorylation, whereas zotatifin treatment caused a reduction in HER3 signaling both as a single agent and in combination with Ipatasertib. This is consistent with the downregulation of HER2 protein levels with zotatifin treatment alone and in combination (**Figure 5B**). In addition, cyclin D1 was downregulated by zotatifin and a reduction in protein levels was maintained in the combination treatment.

Western blot analysis of zotatifin and Ipatasertib treatments in the SK-BR-3 cell line support the idea that the synergistic anti-tumor activity could be attributed to the elimination of RTK induction and reversal of PI3K/AKT feedback relief outcome. To further investigate this idea, we tested the effect of zotatifin and PI3K/AKT combination treatments on additional RTK-driven models. Zotatifin was combined with the PI3K or AKT pathway inhibitors, Alpelisib or Ipatasertib in the OE19 HER2 driven cell line and evaluated by western blot analysis (**Supplementary Figures S5A, B**). Ipatasertib or Alpelisib alone lead to an increase in HER3 signaling whereas zotatifin alone or in combination caused a dose dependent reduction in HER3 signaling, consistent with the down regulation of HER2 protein levels (**Supplementary Figures S5A, B**).

**Figure 6:**
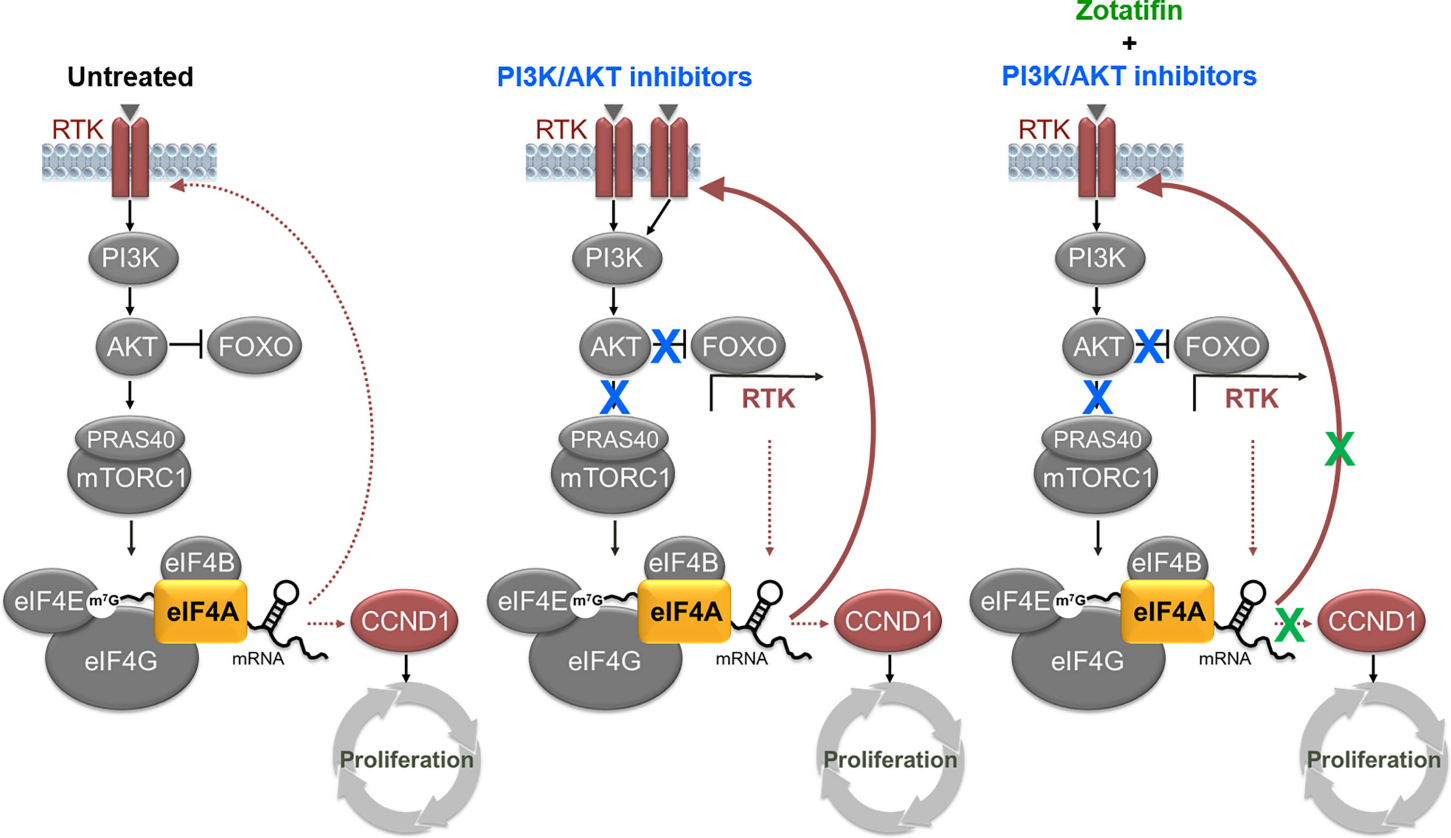
Model for combination benefit of vertical inhibition of PI3K/AKT/eIF4F pathway. Combined downregulation of AKT activity, RTK expression/activation and cyclin D1-dependent proliferation is effective in treatment of RTK-driven tumors. Left, in untreated RTK-driven cancer cells, hyperactivation of eIF4A sustains expression of oncogenic RTKs and cyclin D1. Center, treatments targeted at PI3K/AKT inhibition are limited by relief of feedback loops that induce RTKs activation (20). Right, combination of PI3K/AKT inhibitors with zotatifin reduce undesired RTK activation, while maintaining low AKT activity and low expression of oncogenic cyclin D1.

To further understand the mechanism of combination benefit for the models tested *in vivo*, the effects on signaling pathways were analyzed in the JIMT-1 cell line (HER2^amp^) treated with zotatifin and Alpelisib and the MFM-223 cell line (FGFR2^amp^) treated with zotatifin and Ipatasertib. In both cases, treatment with PI3K/AKT inhibitor alone led to reduced AKT activity, reduced FOXO phosphorylation (increased FOXO activity) and increased HER3 signaling (**Figures 5C, D**). Combination with zotatifin reversed the increased HER3 signaling caused by AKT inhibition resulting in a deeper inhibition of AKT substrates due to down regulation of RTKs (HER2 and FGFR2) as well as downregulating cyclin D1 protein levels (**Figures 5C, D**). Together, this data supports the idea that vertical inhibition of the PI3K/AKT/eIF4F pathway is synergistic in RTK-driven tumor models. We propose that the synergistic effect is attributed to zotatifin-dependent reversal of RTK activation, a well-characterized by product of AKT inhibition (20, 21), along with significant inhibition of tumor cell proliferation (**Figure 6**).

## Discussion

Zotatifin is an eIF4A inhibitor that translationally regulates select target genes by increasing the affinity between eIF4A and polypurine RNA sequence/structural motifs, thereby converting eIF4A into a sequence specific translation repressor. We show that the HER2 and FGFR1/2 RTKs, which contain zotatifin binding motifs in their 5’-UTRs, are downregulated by zotatifin, thus providing the rationale to treat RTK-driven tumors with zotatifin.

Zotatifin demonstrated broad anti-tumor activity across HER2^amp^ or FGFR1/2^amp^ driven cancer cell lines *in vitro* exhibiting both inhibition of tumor cell proliferation and induction of apoptosis. The comprehensive anti-proliferative effect may be attributed to downregulation of the RTK coupled with reduction of the cell cycle regulator cyclin D1 with zotatifin treatment. Significant induction of apoptosis (> 3-fold) was observed in most tumor models following treatment with zotatifin *in vitro*. This *in vitro* anti-tumor activity corresponded to significant *in vivo* tumor growth inhibition and regression in RTK-driven xenograft models treated with zotatifin.

A small subset of the models tested exhibited only limited, if any, induction of apoptosis after 48 h treatment with zotatifin. This subset of models also turned out to be refractory to zotatifin anti-tumor activity *in vivo*. Analysis aimed at deciphering predictors of sensitivity and/or response to zotatifin *in vivo* revealed that the combined average of relative expression levels of several phosphorylation biomarkers (“mTOR activity” score composed of p-mTOR (S2448), p-p70S6K (T389), p-Rictor (T1135), p-rpS6 (S240/244 and S235/236) levels) may predict sensitivity, with a high score being correlated to high sensitivity to zotatifin. The identification of high mTOR activity as a driver of sensitivity suggests that markers of mTOR signaling coupled with RTK dysregulation (amplification, mutation and fusions) can be used as potential markers for patient selection.

Intriguingly, RTK-driven tumors that do not undergo apoptosis with zotatifin treatment exhibit less significant downregulation of p-ERK1/2 T202/Y204 and p-AKT S473 signaling following treatment with zotatifin. The reason for the limited attenuation of p-ERK and p-AKT signaling in non-sensitive cell lines is not clear, given that the driver RTKs are downregulated with zotatifin treatment in both sensitive and non-sensitive models. Limited sensitivity to zotatifin in the subset of RTK-driven tumors implies the presence of bypass signaling pathways that maintain the activation of the PI3K/AKT and RAS/ERK pathways. In fact, bypass signaling comprises known intrinsic and acquired resistance mechanisms to conventional therapies aimed at PI3K or AKT inhibition. Higher sensitivity of some RTK-driven models compared to others may also arise from downregulation of additional oncogenes (*i.e.* cyclin D1 and c-MYC) by zotatifin in the RTK-dependent signaling pathways.

Paradoxically with its significance as a therapeutic target, activation of the PI3K/AKT pathway also confers resistance to conventional therapies aimed at PI3K and AKT inhibition. Unintended compensatory activation of the pathway in response to suppression of these signaling proteins has been associated with several mechanisms, including the upregulation and/or activation of compensatory signaling pathways through alternative RTKs (*e.g*., MET, IGF-1R, FGFR, EphA2) (31) and the FOXO3a-dependent up-regulation of HER3 and increased HER3 signaling output (21, 26).

HER3 is important in oncogenic signaling, particularly in HER2-amplified breast cancers, where HER2 preferentially dimerizes with and phosphorylates HER3 (32). Similarly, in FGFR2-amplified gastric cancer cell lines, FGFR2 activates HER3 (33). Ultimately, HER3 activation functions to preserve the downstream tumorigenic signaling, thus limiting the efficacy of PI3K- or AKT-inhibitors. Our analysis of zotatifin activity in RTK-driven tumors demonstrates that zotatifin not only effectively downregulates HER2 or FGFR1/2, but also downregulates HER3 signaling. We reason that zotatifin-dependent downregulation of HER3 signaling is attributed to the sustained downregulation of HER2, likely compromising HER2-HER3 hetero-dimeric formation and thus HER2-dependent activation of HER3. Accordingly, downregulation of FGFR2 in MFM-223 tumor limits FGFR2-dependent activation of HER3.

We propose that the manifestation of a unique circuitry where, on the one hand, RTK activation promotes eIF4A activation while eIF4A inhibition results in translational downregulation of the RTK creates a context of hypersensitivity to zotatifin treatment that results in significant induction of tumor cell death and tumor regression *in vivo*. This pathway dependency also provides an opportunity for combination therapies aimed at vertical inhibition of the oncogenic PI3K/AKT/eIF4F pathway. Our data supports the idea that this combination strategy could potentially limit the negative outcome associated with compensatory activation by PI3K- or AKT-inhibitors and minimize the risk of resistance. Also, based on the key cellular functions of cyclin D1 in driving cell cycle progression, we further propose that downregulation of cyclin D1 by zotatifin contributes to the strong anti-tumor activity of zotatifin and to the effectiveness of combination treatments with PI3K or AKT inhibitors. Collectively, treatment of HER2 or FGFR1/2 driven tumors with zotatifin as a single agent or in combination with PI3K or AKT inhibitors offers a promising clinical strategy.

## Data Availability Statement

The raw data supporting the conclusions of this article will be made available by the authors, without undue reservation.

## Ethics Statement

All animal studies were carried out in accordance with the guidelines established by the Institutional Animal Care and Use Committee at Explora BioLabs (San Diego, CA, ACUP# EB17-010-033), Crown Biosciences (Beijing, China) or Wuxi AppTec (Shanghai, China).

## Author Contributions

Conception and design: AG-G, NY, JS, CS, JC, and PT. Development of Methodology: AG-G, NY, JS, JC, VG, and CS. Acquisition of data: AG-G, NY, VG, BE, CS, SF, MB, ES, JS, and JC. Analysis and interpretation of data: AG-G, NY, VG, BE, CS, SF, MB, ES, JS, JC, PT, and GC. Writing, review and/or revision of manuscript: PT and AG-G wrote the initial draft and all authors reviewed the manuscript. Administrative, technical or material support: PT, GC, and KW. Study supervision: PT, GC, JS, and KW. All authors contributed to the article and approved the submitted version.

## Funding

The research reported here was funded by eFFECTOR Therapeutics.

## Conflict of Interest

AG-G, NY, VG, BE, CS, JC, SF, MB, ES, JS, GC, KW, and PT were employees and stock option holders of eFFECTOR Therapeutics, Inc. when the studies were performed.

The authors declare that this study received funding from eFFECTOR Therapeutics. The funder had the following involvement with the study: All authors were employees of eFFECTOR Therapeutics when the studies were performed.

## Publisher’s Note

All claims expressed in this article are solely those of the authors and do not necessarily represent those of their affiliated organizations, or those of the publisher, the editors and the reviewers. Any product that may be evaluated in this article, or claim that may be made by its manufacturer, is not guaranteed or endorsed by the publisher.
