# Supplementary material for: Zotatifin, an eIF4A-Selective Inhibitor, Blocks Tumor Growth in Receptor Tyrosine Kinase Driven Tumors
Source: Front Oncol. 2021 Nov 24;11:766298. doi: 10.3389/fonc.2021.766298 (PMC8663026; doi:10.3389/fonc.2021.766298)
Supplement: Supplementary file 1 [file DataSheet_1.docx]

**Supplementary Materials and Methods**

**Overexpression of RTKs (ΔUTR)**

ΔUTR constructs were generated by overexpressing the open reading frames of HER2 (ΔUTR) or FGFR2 (ΔUTR) using lentivirus gene expression system (VectorBuilder, Il, USA) in MDA-MB-361 or SNU-16 cells, respectively. For integration selection, vector transduction was followed by puromycin selection (1.0 ug/ml for 7 days) in MDA-MB-361 cells, or cell sorting in SNU-16. Parental cell lines (MDA-MB-361, SNU-16) or cells overexpressing RTK (ΔUTR) were treated with zotatifin for 24 h. Cell lysates were analyzed on the WES Simple Western system according to the manufacturer’s instructions.

**5’-UTR luciferase reporter assays**

Table S1. 5’UTR sequences

| Gene | 5’UTR Sequence |
| --- | --- |
| FGFR1 | AGATGCAGGGGCGCAAACGCCAAAGGAGACCAGGCTGTAGGAAGAGAAGGGCAGAGCGCCGGACAGCTCGGCCCGCTCCCCGTCCTTTGGGGCCGCGGCTGGGGAACTACAAGGCCCAGCAGGCAGCTGCAGGGGGCGGAGGCGGAGGAGGGACCAGCGCGGGTGGGAGTGAGAGAGCGAGCCCTCGCGCCCCGCCGGCGCATAGCGCTCGGAGCGCTCTTGCGGCCACAGGCGCGGCGTCCTCGGCGGCGGGCGGCAGCTAGCGGGAGCCGGGACGCCGGTGCAGCCGCAGCGCGCGGAGGAACCCGGGTGTGCCGGGAGCTGGGCGGCCACGTCCGGACGGGACCGAGACCCCTCGTAGCGCATTGCGGCGACCTCGCCTTCCCCGGCCGCGAGCGCGCCGCTGCTTGAAAAGCCGCGGAACCCAAGGACTTTTCTCCGGTCCGAGCTCGGGGCGCCCCGCAGGGCGCACGGTACCCGTGCTGCAGTCGGGCACGCCGCGGCGCCGGGGCCTCCGCAGGGCGATGGAGCCCGGTCTGCAAGGAAAGTGAGGCGCCGCCGCTGCGTTCTGGAGGAGGGGGGCACAAGGTCTGGAGACCCCGGGTGGCGGACGGGAGCCCTCCCCCCGCCCCGCCTCCGGGGCACCAGCTCCGGCTCCATTGTTCCCGCCCGGGCTGGAGGCGCCGAGCACCGAGCGCCGCCGGGAGTCGAGCGCCGGCCGCGGAGCTCTTGCGACCCCGCCAGGACCCGAACAGAGCCCGGGGGCGGCGGGCCGGAGCCGGGGACGCGGGCACACGCCCGCTCGCACAAGCCACGGCGGACTCTCCCGAGGCGGAACCTCCACGCCGAGCGAGGGTCAGTTTGAAAAGGAGGATCGAGCTCACTGTGGAGTATCCATGGAGATGTGGAGCCTTGTCACCAACCTCTAACTGCAGAACTGGG |
| FGFR2 | GGCGGCGGCTGGAGGAGAGCGCGGTGGAGAGCCGAGCGGGCGGGCGGCGGGTGCGGAGCGGGCGAGGGAGCGCGCGCGGCCGCCACAAAGCTCGGGCGCCGCGGGGCTGCATGCGGCGTACCTGGCCCGGCGCGGCGACTGCTCTCCGGGCTGGCGGGGGCCGGCCGCGAGCCCCGGGGGCCCCGAGGCCGCAGCTTGCCTGCGCGCTCTGAGCCTTCGCAACTCGCGAGCAAAGTTTGGTGGAGGCAACGCCAAGCCTGAGTCCTTTCTTCCTCTCGTTCCCCAAATCCGAGGGCAGCCCGCGGGCGTCATGCCCGCGCTCCTCCGCAGCCTGGGGTACGCGTGAAGCCCGGGAGGCTTGGCGCCGGCGAAGACCCAAGGACCACTCTTCTGCGTTTGGAGTTGCTCCCCGCAACCCCGGGCTCGTCGCTTTCTCCATCCCGACCCACGCGGGGCGCGGGGACAACACAGGTCGCGGAGGAGCGTTGCCATTCAAGTGACTGCAGCAGCAGCGGCAGCGCCTCGGTTCCTGAGCCCACCGCAGGCTGAAGGCATTGCGCGTAGTCCATGCCCGTAGAGGAAGTGTGCAGATGGGATTAACGTCCACATGGAGATATGGAAGAGGACCGGGGATTGGTACCGTAACC |
| HER2 | GCGCTTGCTCCCAATCACAGGAGAAGGAGGAGGTGGAGGAGGAGGGCTGCTTGAGGAAGTATAAGAATGAAGTTGTGAAGCTGAGATTCCCCTCCATTGGGACCGGAGAAACCAGGGGAGCCCCCCGGGCAGCCGCGCGCCCCTTCCCACGGGGCCCTTTACTGCGCCGCGCGCCCGGCCCCCACCCCTCGCAGCACCCCGCGCCCCGCGCCCTCCCAGCCGGGTCCAGCCGGAGCCATGGGGCCGGAGCCGCAGTGAGCACC |
| TUBA | AGTTCTCACTGAGACCTGTCACCCCGACTCAACGTGAGACGCACCGCCCGGACTCACC |

***In vivo* studies**

All animal studies were carried out in accordance with the guidelines established by the Institutional Animal Care and Use Committee at Explora BioLabs (San Diego, CA, ACUP# EB17-010-033), Crown Biosciences (Beijing, China) or Wuxi AppTec (Shanghai, China). For subcutaneous xenograft studies, mice (6-8-week-old females, 16-24 grams) were implanted with an equal volume (1:1) ratio of tumor cells and Matrigel (BD Biosciences, CA, USA) for tumor development. When the mean tumor size reached ~100-200 mm^3^, the mice were randomized and size-matched into vehicle and treatment groups. Tumor size was measured in length and width with a caliper twice a week. The tumor volume was calculated by the formula LxWxW/2 according to NCI standards. Body weights were collected prior to study start and twice a week during the study. Zotatifin was formulated in 5% dextrose in water (D5W) and immediately dissolved into solution and administered IV Q4D.

NCI-H716 5x10^6^ cells in NOD.SCID, mean tumor initiation size 221 mm^3^; MFM-223 5x10^6^ cells in SCID Beige, mean tumor initiation size 120 mm^3^; NCI-H1581 5x10^6^ cells in NOD.SCID, mean tumor initiation size 105 mm^3^; NCI-N87 1x10^7^ cells in BALB/c nude, mean tumor initiation size 156 mm^3^.

For pharmacodynamic (PD) studies, tumors were excised from animals and snap frozen in liquid nitrogen. Frozen tumor was weighed out and 6x (volume:tumor weight) of 1x Cell Lysis Buffer supplemented with protease and phosphatase inhibitors (MilliporeSigma, CA, USA) was added to the sample in a Lysing Matrix A Tube (ThermoFisher, MA, USA). Samples were homogenized using a Precellys 24 homogenizer (Bertin Technologies, MD, USA) and lysates were clarified by centrifugation. Protein concentrations in cell lysates were quantitated by BCA protein assay and equal amounts of total protein were resolved by SDS-PAGE, immunoblotted with the indicated antibodies, and visualized by LI-COR Odyssey imager.


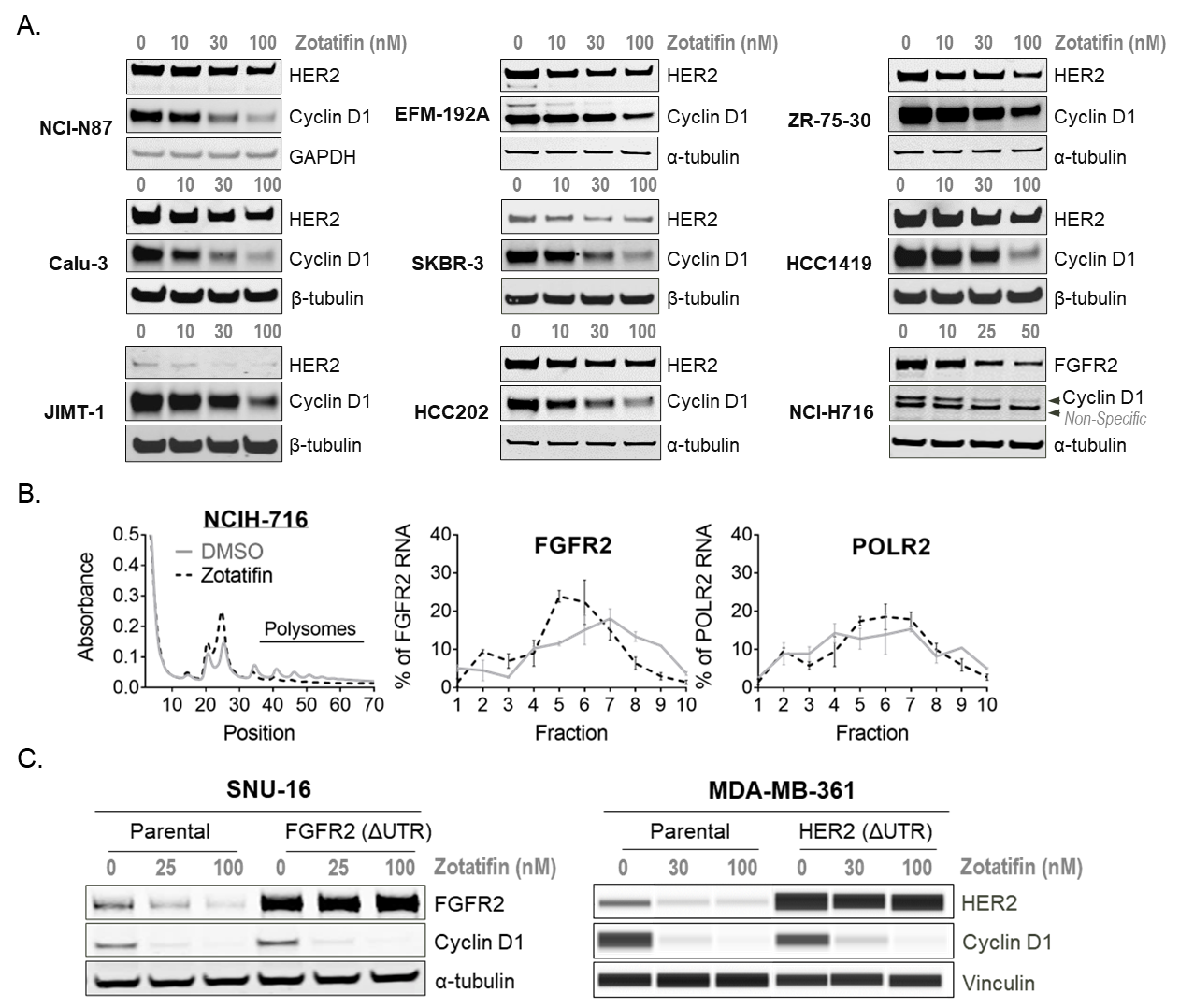


**Supplementary Figure S1: Zotatifin translationally downregulates FGFR2 and HER2 through their 5’-UTRs in RTK-driven tumors.** (A) Western blot analysis of HER2, FGFR2 and cyclin D1 levels following 24 h treatment with zotatifin or DMSO in RTK-driven tumor models. GAPDH, β-tubulin or α-tubulin serve as loading controls. (B) mRNA distribution in polysome fractions in NCI-H716 colorectal cells treated for 3 h with 20 nM zotatifin compared to DMSO. mRNA levels of FGFR2 are monitored in polysome fractions. POLR2 serves as a control. (C) Western blot analysis of the driver RTK and cyclin D1 levels following 24 h treatment with zotatifin or DMSO. Left, parental or FGFR2(ΔUTR)-overexpressed SNU-16 cells. Right, parental or HER2(ΔUTR)-overexpressed MDA-MB-361 cells. α-tubulin or vinculin serve as loading controls.


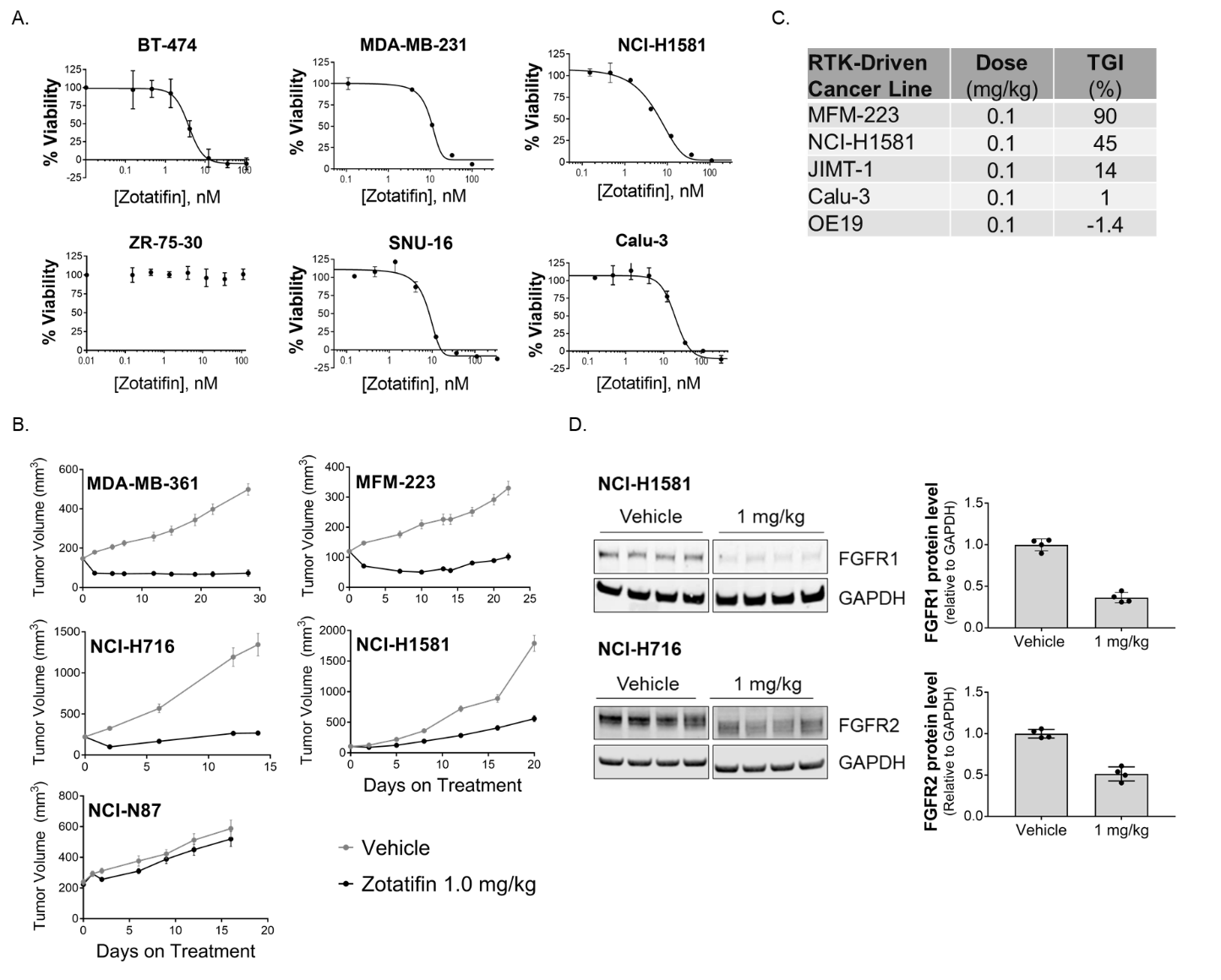
**Supplementary Figure S2: Activity of zotatifin in RTK-driven cancer models.** (A) Dose dependent proliferation curves in the indicated RTK-driven tumor models after 48 h treatment (72 h for Calu-3) with zotatifin *in vitro*. (B) Representative graphs of tumor volume measurement as a function of days of treatment with 1 mg/kg zotatifin. (C) Percent tumor growth inhibition (% TGI) of the subcutaneous tumor in several RTK-driven models, following treatment with 0.1 mg/kg zotatifin (OE19, see also **Figure 2C**). (D) Western blot analysis of FGFR1 in NCI-H1581 (Top) and FGFR2 in NCI-H716 (Bottom) xenografts 24 h post the last dose following treatment with 1 mg/kg zotatifin or vehicle. GAPDH serves as loading control. Right, quantification of FGFR1/2 protein levels.

**
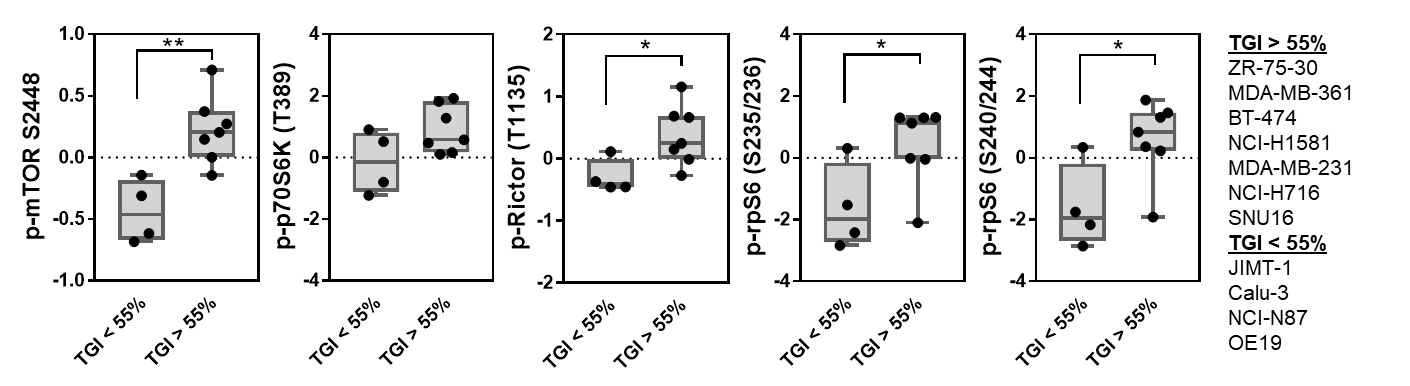
**

**Supplementary Figure S3: Components of “mTOR activity” score.** CCLE-based distribution scores of p-mTOR S2448, p-P70S6K T389, p-Rictor T1135, p-rpS6 S240/244 and p-rpS6 S235/236 in zotatifin-sensitive (> 55% TGI) versus non-sensitive (< 55% TGI) RTK-driven models (see % TGI in **Figure 2C**). The mTOR activity score was calculated by taking the mean of each normalized phosphoprotein score.

**
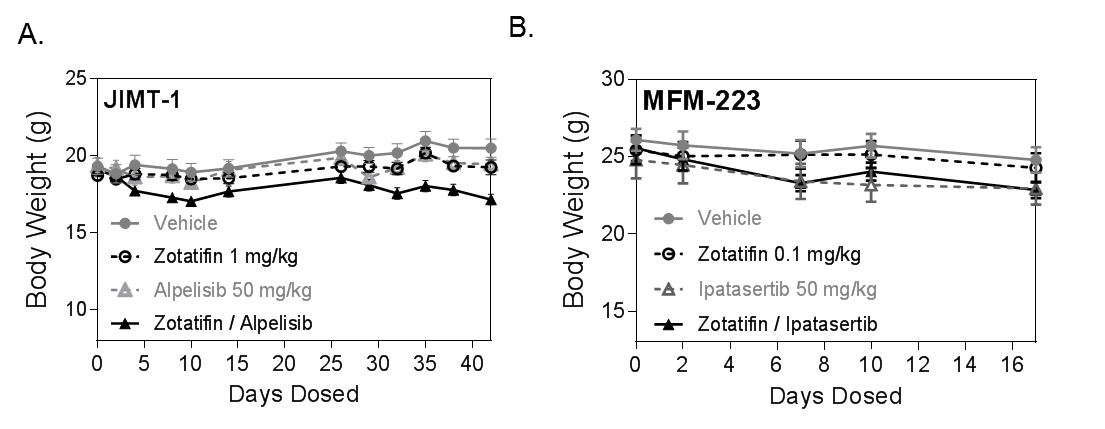
**

**Supplementary Figure S4: Combination treatments in xenografts are well tolerated**. A-B) Body weight of treated mice during the course of treatment in (A) JIMT-1 and (B) MFM-223 xenografts.


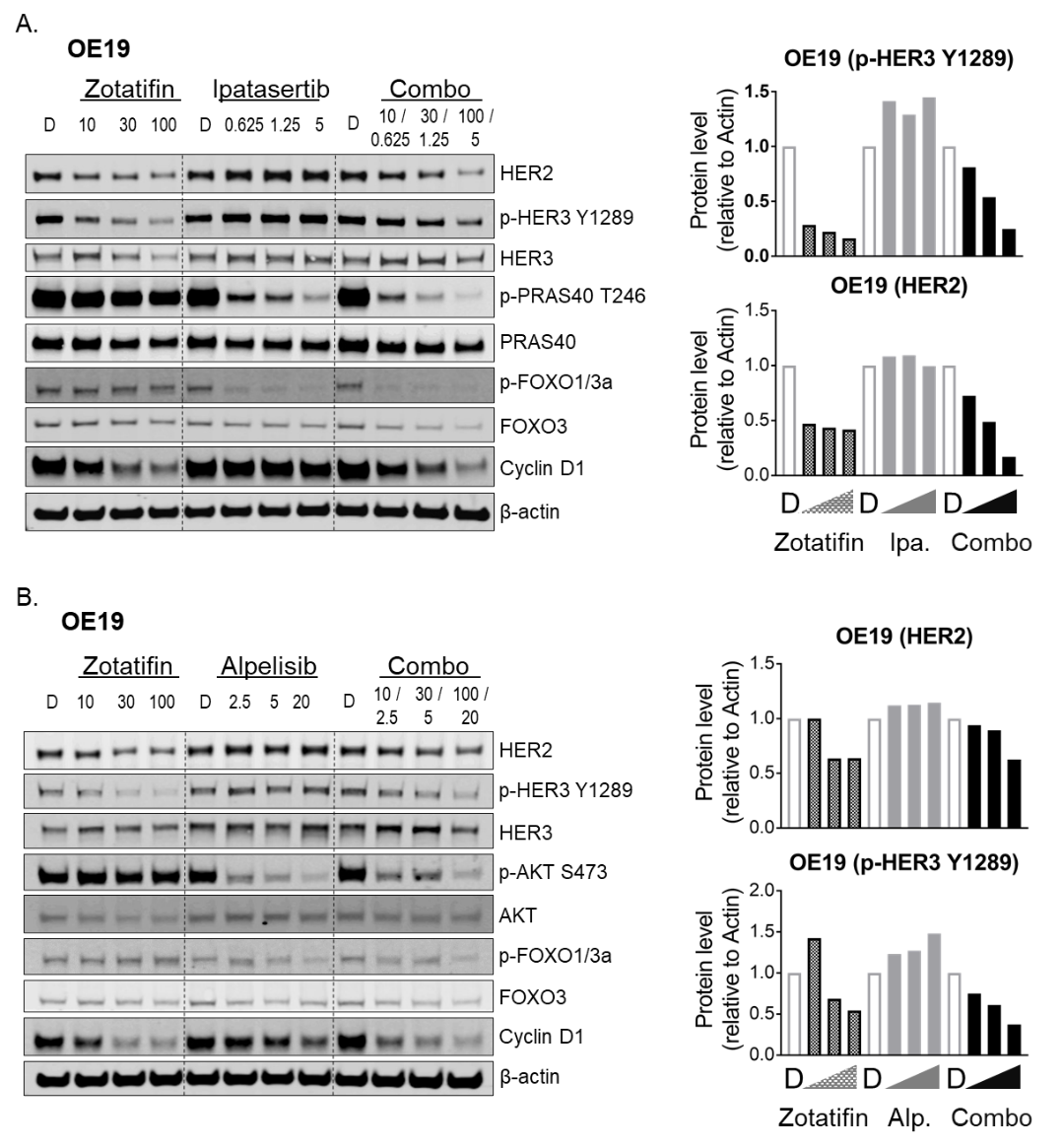


**Supplementary Figure S5: Zotatifin downregulates RTK levels induced by PI3K/AKT inhibition *in vitro*.** (A-B) Western blots analysis of HER3, AKT, PRAS40, FOXO3 (phospho/total), HER2 and cyclin D1 in RTK-driven model following 24 h treatment with either single agent or combinations. (A) OE19 HER2^amp^ cell line treated with zotatifin and Ipatasertib (AKTi); (B) OE19 cell line treated with zotatifin and Alpelisib (PIK3CAi). To the right of each panel, quantification of p-HER3 Y1289 or HER2 proteins levels. Concentrations of drugs used are indicated on top of each blot: Ipa., Ipatasertib (µM); Alp., Alpelisib (µM); zotatifin (nM); D, DMSO.
